# Evidence Based Tailored Parotidectomy in Treating External Auditory Canal Carcinoma

**DOI:** 10.1038/s41598-018-30536-0

**Published:** 2018-08-14

**Authors:** Jeon Mi Lee, Jin Woo Joo, Sung Huhn Kim, Jae Young Choi, In Seok Moon

**Affiliations:** 1 Department of Otorhinolaryngology, Ilsan Paik Hospital, Inje Universtiy College of Medicine, Goyang, Republic of Korea; 2 Department of Pathology, Yonsei University College of Medicine, Seoul, Republic of Korea; 3 Department of Otorhinolaryngology, Yonsei University College of Medicine, Seoul, Republic of Korea

## Abstract

Carcinoma of the external auditory canal (EAC) is a rare tumor and little information is available regarding parotid gland in surgically treating EAC carcinomas. This study aimed to investigate the mode of parotid involvement in EAC carcinoma through staging and histopathological analysis, and to establish surgical guidelines for the parotid gland management when there is no clinical evidence of parotid involvement. Sixty-five patients with EAC carcinoma who underwent temporal bone resection and any type of parotidectomy simultaneously were retrospectively reviewed. The rate of direct parotid invasion and parotid nodal involvement was analyzed according to the stage and histopathological findings. Among the 65 patients, 39 were confirmed to have squamous cell carcinoma (SCC) and 26 were confirmed to have adenoid cystic carcinoma (ACC). Direct parotid invasion occurred in 7 of 39 patients with SCC, only in the advanced stages, and in 15 of 26 patients with ACC, regardless of stage. Metastasis to the parotid node was noted in 6 patients with advanced-stage SCC, whereas no patient with ACC showed parotid nodal metastasis. For adequate tumor control with low risk of surgical complications, evidence based tailored parotidectomy should be applied. With no evidence of parotid involvement, an elective parotidectomy can be excluded in early SCC, whereas a total parotidectomy is recommended for advanced SCC. In ACC, basal resection of the parotid gland rather than a superficial or total parotidectomy should be performed at all disease stages.

## Introduction

Carcinoma of the external auditory canal (EAC) is extremely rare, with an annual incidence of 1 per 1 million population^1^, and it can be life threatening without early aggressive treatment^2^. Various treatments have been considered and studied, and *en bloc* resection of the tumor with an adequate safe margin remains the basic and most important principle^3,4^. However, deciding the extent of resection is problematic, because minimal interventions threaten oncologic safety and aggressive surgical intervention is associated with a higher rate of complications^5^.

Parotidectomy is another concern. Tumors arising from the temporal bone often directly invade the parotid gland through the Santorini fissure or the foramen of Huschke^6^. Furthermore, the parotid gland contains the first lymph nodes drained from the EAC that may indicate that either the parotid gland or the parotid node could be involved in EAC carcinoma^7^. Owing to the rarity of EAC carcinoma, the available literature reports do not provide sufficient information about the mode of parotid involvement in this cancer. There should be no hesitation in performing a therapeutic parotidectomy when parotid involvement is apparent, but performing an elective parotidectomy with no clinical evidence of parotid involvement remains controversial. Some studies reported that there was no correlation between performing elective parotidectomy and the patient survival rate^1,8^, whereas others advocate elective parotidectomy in every cases^9,10^. As parotidectomy has a risk of facial nerve injury and could cause cosmetic issues such as asymmetric facial volume after surgery, unnecessary parotidectomy should be avoided. However, if parotidectomy is omitted in situations in which it is essential, the possibility for recurrence and final mortality highly increases. This dilemma arouses the necessity for proper guidelines for elective parotidectomy in the treatment of EAC carcinoma.

This study was performed to provide guidelines for the management of the parotid gland in treating EAC carcinoma when there is no clinical evidence of parotid involvement. The rate of direct parotid gland invasion and parotid node metastasis in patients with squamous cell carcinoma (SCC) and adenoid cystic carcinoma (ACC) of the EAC was examined according to the postoperative tumor stage. The data include those from a previous report from our institute^11^.

## Results

### Patient Characteristics

There were 6 stage I patients, 8 stage II patients, 8 stage III patients, and 17 stage IV patients in the SCC group. In the ACC group, there were 3 stage I patients, 6 stage II patients, 4 stage III patients, and 13 stage IV patients. The surgical procedures performed were as follows: lateral temporal bone resection (LTBR) in 35 patients with stage I, II, and III; subtotal temporal bone resection in 29 patients with stage III and IV; and total temporal bone resection in 1 patient with stage IV. The details of the patients are listed in Table 1.

**Table 1:** Characteristics of the Patients.

Patient No.	Sex/Age (Years)	Preoperative Stage	Surgery	Parotidectomy	Neck Dissection
**Patients with Squamous Cell Carcinoma**					
1	F/68	I	LTBR	Superficial	None
2	M/73	I	LTBR	Total	Lv. I, II, III, IV, V
3	M/79	I	LTBR	Superficial	None
4	M/60	I	LTBR	Total	Lv. I, II, III
5	M/37	I	LTBR	Superficial	None
6	M/64	I	LTBR	Total	Lv. I, II, III
7	M/34	II	LTBR	Superficial	None
8	F/41	II	LTBR	Superficial	None
9	F/79	II	LTBR	Superficial	None
10	F/64	II	LTBR	Superficial	Lv. II
11	F/52	II	LTBR	Superficial	None
12	M/67	II	LTBR	Superficial	None
13	F/67	II	LTBR	Superficial	None
14	F/60	II	LTBR	Superficial	None
15	M/70	III	LTBR	Superficial	None
16	M/68	III	LTBR	Partial	None
17	M/66	III	LTBR	Superficial	None
18	F/65	III	STBR	Total	Lv. II
19	M/60	III	STBR	Total	Lv. II
20	M/57	III	STBR	Total	None
21	F/64	III	STBR	Total	Undescribed
22	F/49	III	STBR	Total	Undescribed
23	F/49	IV	LTBR	Partial	None
24	F/72	IV	LTBR	Superficial	None
25	M/48	IV	LTBR	Total	Lv. III
26	F/54	IV	LTBR	Superficial	None
27	M/70	IV	LTBR	Superficial	Lv. II, III
28	M/43	IV	LTBR, auricle resection	Total	Lv. I, II, III, IV, V
29	F/76	IV	STBR	Superficial	None
30	M/52	IV	STBR	Total	None
31	M/47	IV	STBR	Total	Lv. I
32	F/55	IV	STBR	Total	Lv. IV
33	F/44	IV	STBR	Total	None
34	M/45	IV	STBR	Total	Lv. II
35	M/58	IV	STBR	Superficial	Lv. I, II, III
36	M/56	IV	STBR	Total	Undescribed
37	M/62	IV	STBR	Total	Undescribed
38	M/48	IV	STBR	Total	Undescribed
39	M/35	IV	TTBR	Total	Lv. I, II, III
**Patients with Adenoid Cystic Carcinoma**					
1	M/57	I	LTBR	Total	None
2	F/47	I	LTBR	Superficial	None
3	F/49	I	LTBR	Superficial	None
4	M/66	II	LTBR	Superficial	None
5	F/44	II	LTBR	Superficial	None
6	F/38	II	LTBR	Superficial	None
7	F/71	II	LTBR	Superficial	None
8	M/52	II	STBR	Superficial	None
9	F/50	II	STBR	Superficial	None
10	F/48	III	LTBR	Superficial	None
11	F/47	III	LTBR	Superficial	None
12	F/29	III	LTBR	Superficial	None
13	F/60	III	STBR	Total	Undescribed
14	M/50	III	STBR	Total	Undescribed
15	F/53	IV	LTBR	Total	None
16	M/51	IV	LTBR	Total	None
17	F/54	IV	STBR	Superficial	Lv. II
18	F/49	IV	STBR	Superficial	None
19	F/67	IV	STBR	Superficial	Lv. II
20	F/47	IV	STBR	Total	None
21	M/45	IV	STBR	Total	None
22	M/26	IV	STBR	Total	None
23	M/33	IV	STBR	Total	None
24	F/60	IV	STBR	Total	Undescribed
25	F/67	IV	STBR	Total	Undescribed
26	F/53	IV	STBR	Total	Undescribed

LTBR = lateral temporal bone resection; STBR = subtotal temporal bone resection; TTBR = total temporal bone resection; Lv. = level.

### Direct Parotid Invasion Pattern

**Figure 1:**
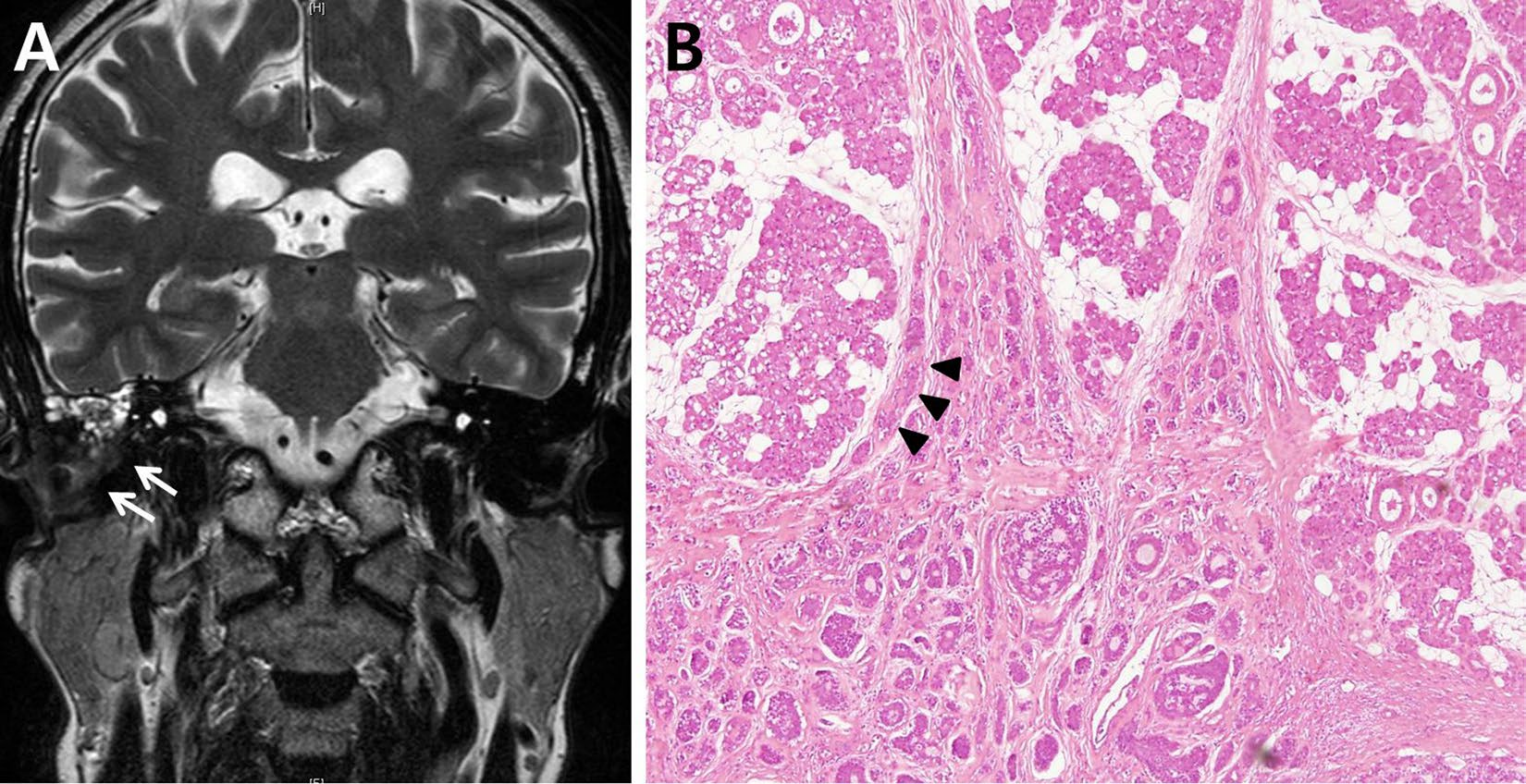
Parotid involvement in adenoid cystic carcinoma. (**A**) Magnetic resonance image demonstrating the findings of external auditory canal (EAC) carcinomas (white arrows). The tumor is confined in the EAC without parotid involvement. (**B**) Histopathologic specimen from the same patients. The tumor is directly extending through the cartilage and an invading lesion in the parotid gland (black arrow head) is shown (magnification, x100).

In SCC, direct parotid invasion was noted only in advanced-stage cases (two at stage III and five at stage IV). In ACC, direct parotid invasion was more common, and invasion was observed even in early-stage cases (two at stage I, three at stage II, two at stage III, and eight at stage IV; Fig. 1). Direct parotid invasion in early carcinoma was significantly more common in ACC than in SCC (*p* = 0.004, Table 2). Direct parotid invasion was also more common in advanced SCC than in early SCC (*p* = 0.036, Table 2).

**Table 2:** Pattern of Parotid Involvement in External Auditory Canal Carcinoma According to Stage and Pathology.

Stage		Squamous Cell Carcinoma		Adenoid Cystic Carcinoma		P-value
		Patients (n)	Direct Invasion (%)	Patients (n)	Direct Invasion (%)	
Early-stage	I	6	0 (0%)	3	2 (67%)	0.004
	II	8	0 (0%)	6	3 (50%)	
Advanced-stage	III	8	2 (25%)	4	2 (50%)	0.061
	IV	17	5 (29%)	13	8 (62%)	
P-value			0.036		1.000	

### Parotid Nodal Metastasis Pattern

Seven cases of SCC at an advanced stage (two at stage III and four at stage IV) had subclinical parotid nodal metastasis, but none of the patients with ACC had parotid nodal metastasis (Table 3). Although the differences were not significant (*p* = 0.071 and *p* = 0.066), probably due to the small numbers of patients, parotid node metastasis was more common in advanced SCC than in early or advanced ACC.

**Table 3:** Pattern of Node Metastasis in External Auditory Canal Carcinoma According to Stage and Pathology.

Stage		Squamous Cell Carcinoma		Adenoid Cystic Carcinoma		P-value
		Patients (n)	Node Metastasis (%)	Patients (n)	Node Metastasis (%)	
Early-stage	I	6	0 (0%)	3	0 (0%)	1.000
	II	8	0 (0%)	6	0 (0%)	
Advanced-stage	III	8	2 (25%)	4	0 (0%)	0.066
	IV	17	4 (24%)	13	0 (0%)	
P-value			0.071		1.000	

## Discussion

Primary malignant tumors of the EAC are rare, with an incidence of <1 per 1 million population per year^1^. SCC is the most common type of EAC carcinoma, accounting for 80% of EAC tumors, whereas ACC accounts for 5% as the second most common type^12^. SCC and ACC have their own characteristics; but owing to the rarity of EAC carcinoma, their characteristic differences in this cancer are not clearly specified. In this study, we performed a retrospective review of surgical specimens, and we found that direct parotid invasion occurred only in advanced-staged SCC, whereas ACC showed direct parotid invasion both in the early and advanced stages. SCC is known to involve both the cartilaginous and bony parts of the EAC, whereas ACC tends to involve only the cartilaginous part in the early stage^13^. The Santorini fissure, a potent outlet to the parotid gland, is located at the anterior part of the cartilage of the EAC. The tendency of ACC to grow on the superficial part of the EAC and the anatomical position of the Santorini fissure may explain the frequent direct invasion of ACC to the parotid gland even in the early stage. Huschke’s foramen is another outlet to the parotid gland and located at the floor of the bony part of the EAC. Since it is normally closed in adults^14^, it could be ignored in the general population. In case of SCC, the carcinoma is completely confined to the bony tube and thus is relatively not capable of invading the parotid gland directly. However, the growth pattern in the advanced stage, such as bony structure or soft tissue invasion, showed no significant difference between the two histopathologies^13^.

The parotid gland area is abundantly supplied by various nearby lymph nodes, with the lymphatic drainage from the facial skin and scalp being the most representative. The EAC skin also has a lymphatic drainage to the parotid gland. While most external ear carcinomas show parotid node involvement, EAC carcinoma involves only the parotid node when it is extensive and in the advanced stages^15,16^. The causes for this difference are not well known, but Choi *et al*. suggested that the lymphatic network of the EAC might be less developed than that in the external ear^11^. Our study revealed node involvement in only advanced-stage SCC cases, as shown in previous reports. ACC showed no nodal involvement in all stages in our study, and this result was also identical to the report by Xia *et al*.^13^. The cause of the difference is thought to be the histopathologic characteristics. The characteristics of ACC of the EAC are known to be as same as those encountered in small salivary gland ACC, including silent growth, local recurrence, perineural invasion rather than nodal involvement, and late distal metastasis, mostly to the lung, even in the early stages^17^.

Although all patients in our study received parotidectomy, either therapeutic or elective, treatment failure such as tumor recurrence or distant metastasis also occurred. However, it cannot be concluded that parotidectomy is not necessary. Ihler *et al*. studied 36 patients with EAC carcinoma and reported that the patients without parotidectomy showed a mean survival time below the average of the total group^3^. Leong *et al*. studied 35 patients with SCC of the EAC^8^, and although parotidectomy was not statistically considered a prognostic factor in their report, they recommended elective parotidectomy for all T3 and T4 cancers for best practice. Also, Zhang et al. suggested that superficial parotidectomy is necessary for patients with early-stage EAC carcinoma, as well as for those with advanced stages^4^. Parotidectomy is undoubtedly required in the surgical treatment of EAC carcinoma in some patients, but the proper extension of parotidectomy is still controversial. Moreover, although the complication rates after parotidectomy are not very high^18^, critical complications such as facial nerve injury, first bite syndrome, Frey syndrome, or facial asymmetry definitely lower the quality of life^19–21^.

**Figure 2:**
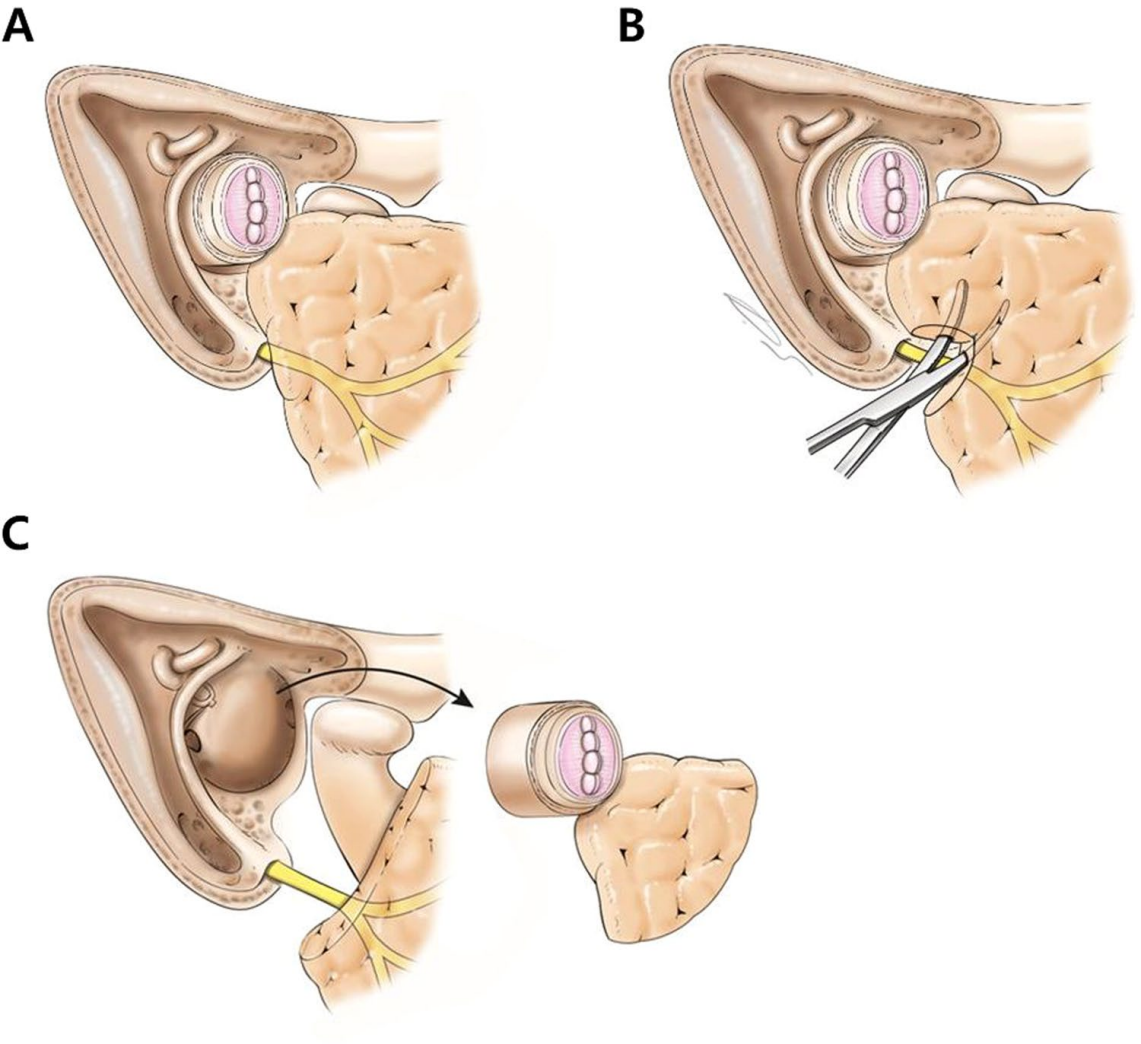
Schematic drawing of the suggested partial basal resection of the parotid gland during temporal bone resection in treating adenoid cystic carcinoma. (**A**) Removal of the bone from 360° around the canal and isolation of the ear canal as an *en bloc* specimen. (**B**) Resection of the gland abutting the ear canal to include it in the specimen. (**C**) Lateral temporal bone resection with a partial basal resection of the parotid gland in continuity with the ear canal.

Our data indicated that elective total parotidectomy to secure an adequate safety margin and to control occult parotid node metastasis is mandatory for advanced-stage SCC, even when there is no clinical evidence for parotid involvement. However, in terms of the characteristics and mode of parotid involvement in ACC, elective total parotidectomy is somehow an excessive treatment for this tumor. Instead, partial resection of the basal (postero-superior) part of parotid gland can provide an adequate safety margin in treating ACC, which can be easily applied while performing temporal bone resection (Fig. 2). Simple resection of the gland abutting the ear canal that lies posterior to the upper branch of the facial nerve could achieve oncological benefits in ACC. This partial basal resection strategy is free from additional incision lines and critical complications after parotidectomy, as described above. The operation time is also reduced. Nevertheless, a demarcation of the facial nerve to a certain degree is also required in partial resection. The known factors associated with a higher incidence of facial nerve deficit include the extent of surgery and the stretching and sectioning of facial nerve branches during surgery^22^. It can be interpreted that manipulating the facial nerve causes facial nerve deficit. The suggested partial basal resection strategy requires a demarcation of the facial nerve without the need for manipulating it; therefore, safer outcomes are expected. The extent of resection should be considered. If parotid infiltration is not detected in preoperative imaging evaluations, considering their detectable limitations, we suggest that the proper surgical margin should be about 1 cm.

Perineural invasion should be considered in treating ACC of the EAC carcinoma because it is a common histological finding in ACC and is a possible route for tumor cell dissemination^23^. However, the presence of perineural invasion is known to be an adverse prognostic factor only when a major nerve is involved^24^, which can be detected preoperatively. In such cases, radical parotidectomy including the facial nerve is essential for oncological safety and reducing mortality. Otherwise, if a major nerve is not involved, there is no evidence that partial resection of the parotid gland rather than parotidectomy increases the rate of perineural invasion. Parotidectomy need not be performed to control preoperatively undetected perineural invasion, and a partial resection of the parotid gland is thought to be a sufficient treatment for EAC carcinoma with ACC. For suspected microscopic perineural invasion, postoperative radiation could be applied.

For adequate tumor control with low risk of surgical complications, evidence based tailored parotidectomy should be applied. A therapeutic parotidectomy must be performed when there is clinical evidence of parotid involvement. However, with no evidence of parotid involvement, an elective parotidectomy can be excluded in early SCC, whereas a total parotidectomy is recommended for advanced SCC. In ACC, basal resection of the parotid gland rather than a superficial or total parotidectomy should be performed at all disease stages. Since treatment is predicated on the underlying pathology and tumor extent as defined by imaging studies, a prudent preoperative evaluation is essential.

There is a critical limitation to the suggested strategy from actual applications. Practical data or supporting references are hardly available. Tumor control results from none, partial and total resection of parotid gland in treating early-stage ACC should be compared to prove the oncological safety and the benefits. To our knowledge, one paper compares the surgical outcomes of early ACC using different surgical procedures. In that study, eight patients underwent LTBR only and nine underwent LTBR including superficial parotidectomy. The 5-year survival rate for the LTBR-only group was 75%, whereas the LTBR plus superficial parotidectomy group had a 100% survival rate. The results underscore the reliability of the suggested strategy. Nevertheless, multicenter retrospective chart reviews or meta-analyses of EAC carcinoma with various parotidectomy procedures are required to strengthen our conclusions.

## Methods

### Patients

The study included 65 patients (31 males, 34 females; mean age, 55 years; age range, 26–79 years) with primary EAC carcinoma, undergoing parotidectomy with various types of temporal bone resection between August 1989 and October 2015 at the Department of Otorhinolaryngology, Yonsei University. Histopathological examination confirmed 39 cases of SCC and 26 cases of ACC. The cancer stages were determined preoperatively through radiographic evaluations such as computed tomography and magnetic resonance imaging, and postoperatively with pathologic confirmation according to the modified Pittsburgh tumor staging system^15^. Preoperative images were interpreted by blinded two neuroradiologists with special regard to the origin of the carcinoma. A retrospective review of the surgical specimens was performed with specific reference to parotid node metastasis and parotid invasion according to the tumor stage and pathology.

All procedures were in accordance with the Declaration of Helsinki of 1975. The study was approved by the IRB of Severance Hospital, Seoul, Korea (4-2018-0060).

### Treatment

Surgery was carried out as an initial treatment in all patients. The procedures have been described in detail by Gacek and Goodman^7^. Postoperative radiation, adjuvant chemotherapy, or concurrent chemoradiation was applied on the basis of pathologic stage, surgical margin status, or the presence of perineural invasion in some advanced-staged cases. All patients in our study underwent therapeutic or elective parotidectomy involving partial, superficial, or total resection.

## Data Availability

The datasets generated during and/or analysed during the current study are available from the corresponding author on reasonable request.
